# Correction: The genomic basis of mood instability: identification of 46 loci in 363,705 UK Biobank participants, genetic correlation with psychiatric disorders, and association with gene expression and function

**DOI:** 10.1038/s41380-019-0466-5

**Published:** 2019-07-30

**Authors:** Joey Ward, Elizabeth M. Tunbridge, Cynthia Sandor, Laura M. Lyall, Amy Ferguson, Rona J. Strawbridge, Donald M. Lyall, Breda Cullen, Nicholas Graham, Keira J. A. Johnston, Caleb Webber, Valentina Escott-Price, Michael O’Donovan, Jill P. Pell, Mark E. S. Bailey, Paul J. Harrison, Daniel J. Smith

**Affiliations:** 1 Institute of Health and Wellbeing, University of Glasgow, Glasgow, UK; 2 Department of Psychiatry, University of Oxford, Oxford, UK; 3 Oxford Health NHS Foundation Trust, Oxford, UK; 4 UK Dementia Research Institute, Cardiff University, Cardiff, UK; 5 Department of Medicine Solna, Karolinska Institute, Stockholm, Sweden; 6 Department of Physiology, Anatomy and Genetics, Oxford, UK; 7 University of Cardiff, Cardiff, UK; 8 School of Life Sciences, College of Medical, Veterinary and Life Sciences, University of Glasgow, Glasgow, UK

**Keywords:** Genetics, Neuroscience

**Correction to: Molecular Psychiatry**

10.1038/s41380-019-0439-8 published online 05 June 2019

This article was originally published under standard licence, but has now been made available under a [CC BY 4.0] license. The PDF and HTML versions of the paper have been modified accordingly.

